# Doxorubicin-Mediated Bone Loss in Breast Cancer Bone Metastases Is Driven by an Interplay between Oxidative Stress and Induction of TGFβ

**DOI:** 10.1371/journal.pone.0078043

**Published:** 2013-10-30

**Authors:** Tapasi Rana, Anwesa Chakrabarti, Michael Freeman, Swati Biswas

**Affiliations:** 1 Department of Radiation Oncology, Vanderbilt University School of Medicine, Nashville, Tennessee, United States of America; 2 Department of Cancer Biology, Vanderbilt University School of Medicine, Nashville, Tennessee, United States of America; 3 College of Pharmacy, University of Tennessee Health Science Center, Memphis, Tennessee, United States of America; Wayne State University School of Medicine, United States of America

## Abstract

Breast cancer patients, who are already at increased risk of developing bone metastases and osteolytic bone damage, are often treated with doxorubicin. Unfortunately, doxorubicin has been reported to induce damage to bone. Moreover, we have previously reported that doxorubicin treatment increases circulating levels of TGFβ in murine pre-clinical models. TGFβ has been implicated in promoting osteolytic bone damage, a consequence of increased osteoclast-mediated resorption and suppression of osteoblast differentiation. Therefore, we hypothesized that in a preclinical breast cancer bone metastasis model, administration of doxorubicin would accelerate bone loss in a TGFβ-mediated manner. Administration of doxorubicin to 4T1 tumor-bearing mice produced an eightfold increase in osteolytic lesion areas compared untreated tumor-bearing mice (*P* = 0.002) and an almost 50% decrease in trabecular bone volume expressed in BV/TV (*P* = 0.0005), both of which were rescued by anti-TGFβ antibody (1D11). Doxorubicin, which is a known inducer of oxidative stress, decreased osteoblast survival and differentiation, which was rescued by N-acetyl cysteine (NAC). Furthermore, doxorubicin treatment decreased Cu-ZnSOD (SOD1) expression and enzyme activity *in vitro*, and treatment with anti-TGFβ antibody was able to rescue both. In conclusion, a combination therapy using doxorubicin and anti-TGFβ antibody might be beneficial for preventing therapy-related bone loss in cancer patients.

## Introduction

More than one million new cases of breast cancer are diagnosed worldwide annually. A major clinical complication of metastatic breast cancer is osteolytic bone destruction, and more than 75% of patients suffer from osteolysis, leading to increased fracture risk and poor quality of life. The overall life expectancy of breast cancer patients has improved in recent years, thereby increasing the patient population that suffers from cancer-induced bone loss, pathological fracture, spinal cord compression, bone pain and hypocalcaemia [1]. In addition, breast cancer patients often experience low bone mineral density and accelerated bone loss as an unavoidable side effect of cancer therapies [2]. While local bone resorption can be induced by the cancer cells at the metastatic site, commonly used cancer therapies often cause additional rapid and systemic bone loss, posing a major challenge in the long term management of patients with breast cancer.

Doxorubicin, an anthracycline agent, is widely used in chemotherapy regimens to treat both early stage and late stage metastatic breast cancer patients [3]. Multiple lines of evidence demonstrate several adverse effects of doxorubicin on bone. Childhood recipients of doxorubicin suffer long term bone damage in the form of reduced adult height and increased fracture risk [4]. A recent study indicated that premenopausal breast cancer patients treated with a doxorubicin/cyclophosphamide combination exhibited low bone mineral density and significant bone loss [5], suggesting a cause and effect relationship between doxorubicin treatment and systemic bone loss. Doxorubicin exposure caused a 60% reduction in bone formation in normal rats, suggesting a potential for reduced osteoblast differentiation [6], [7]. Doxorubicin has also been shown to negatively regulate trabecular bone volume and cortical bone thickness in rabbits [8]. At a cellular level, doxorubicin has been reported to inhibit cell proliferation and parameters of cell differentiation in MC3T3 mouse osteoblasts [9]. Interestingly, Buttiglieri et al. have recently reported that doxorubicin treatment in cultured human mesenchymal stem cells reduced clonogenic ability and decreased osteoblast differentiation, accompanied by increased adipogenic potential [10]. These adverse effects heighten the concern for breast cancer patients who have received doxorubicin as a part of their treatment regimen, because these patients are already at high risk for bone loss and pathological fracture.

Thus far, the cellular and molecular mechanisms of doxorubicin-mediated bone loss are not known in the context of breast-to-bone metastases. In this regard, we have previously reported that doxorubicin exposure increases circulating levels of transforming growth factor-β (TGFβ) in normal and tumor-bearing mice [11]. In addition, Li et al. have reported that doxorubicin treatment can activate TGFβ/Smad signaling pathways [12]. TGFβ is sequestered throughout the skeletal system in its inactive form, making bone the largest source of TGFβ in the human body. TGFβ has been reported to increase osteoclast differentiation and suppress osteoblast differentiation [13], both of which may contribute to pathologic bone loss. TGFβ may also regulate the biochemical properties of bone by controlling bone mass and bone matrix properties [14], and genetic manipulation of this pathway affects bone mass in murine models [15], [16], [17], [18]. Increased TGFβ has been implicated in bone fragility and osteoporosis [16]. We and others have reported that blocking excess TGFβ in the bone, either by antibodies [19], [20] or by small molecule inhibitors [21], is favorable for bone formation. Increased TGFβ has been implicated in induction of reactive oxygen species (ROS) [22], which also agrees with the fact that doxorubicin increases oxidative stress. Several reports have indicated that increased oxidative stress negatively impacts bone formation by modulating the differentiation and survival of osteoblasts [23], [24] and by increasing bone resorption [25], [26]. In addition, ROS accumulation and dysregulation of the antioxidant system are shown to be important mediators of bone loss [27], [28], [29], [30]. Based on these, we hypothesized administration of doxorubicin would accelerate bone loss in a TGF-β-mediated manner.

In this study, we aim to determine the cellular and molecular mechanism of doxorubicin-mediated bone injury in preclinical models of breast cancer to bone metastases. Our data demonstrate, co-administration of doxorubicin with 1D11, a pan-TGFβ antibody that blocks all three isoforms of TGFβ, significantly increased bone volume and decreased osteolytic bone damage. Interestingly, in MC3T3 osteoblast cells, treatment with doxorubicin was able to decrease the level of SOD1, a cytosolic antioxidant enzyme which plays an active role in protecting against oxidative stress by conversion of superoxide radical to hydrogen peroxide [31], [32]. Overproduction of ROS has been implicated in bone resorption [26], [33], osteoporosis and bone fragility [24], [34], [35]. Furthermore, treatment with doxorubicin also inhibited SOD1 enzyme activity in MC3T3 cells. Data presented here indicate treatment with anti-TGFβ antibody was able to restore both SOD1 expression and enzyme activity. Taken together our finding suggests that doxorubicin treatment directly contributes to bone damage by increasing oxidative stress and decreasing SOD1 expression and activity, likely via a TGFβ-mediated process. To our knowledge, it is the first report of doxorubicin mediated bone damage in preclinical breast cancer models.

## Results

### Doxorubicin Treatment Increases Bone Loss in Non-tumor and Tumor Bearing Mice

**Figure 1:**
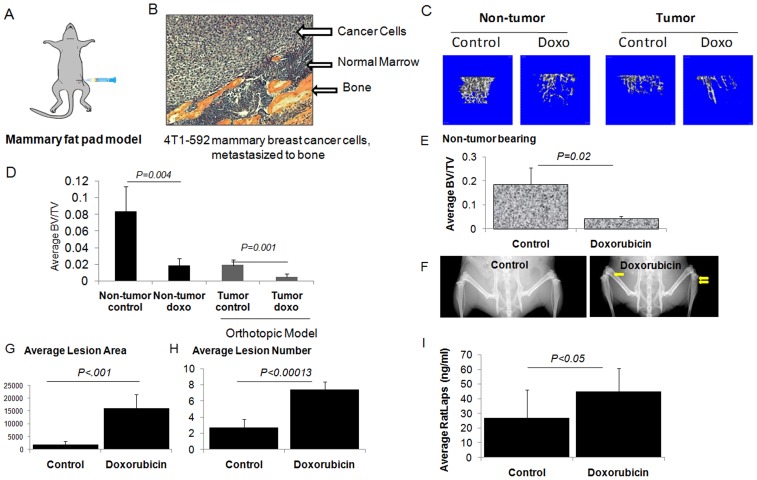
Doxorubicin treatment accelerates bone loss in preclinical breast cancer bone metastasis model. Five-week old female Balb/C mice received either 5 mg/Kg doxorubicin (i.p. once weekly for three weeks) or PBS and microCT of tibiae were performed. For orthotopic breast cancer bone metastasis model, another set of four-week old female Balb/C mice were injected in the left #4 mammary fat pad using a bone-tropic 4T1 cell line and upon tumor development (palpable size after 1 week) mice were treated with either PBS or doxorubicin (5 mg/Kg doxorubicin, i.p., once weekly for three weeks) and microCT and histology of tibiae were performed. (A) Schematic representation of orthotopic injection of 4T1 cells in mammary fat pad. (B) Histology of mouse tibia showing tumor cells in the bone. (C) Representative microCT images (3D reconstruction) of tibiae collected from non-tumor bearing and tumor-bearing Balb/C mice after three weeks of treatment and (D) Quantification of average BV/TV showing further decrease in trabecular bone volume in tumor-bearing mice upon doxorubicin treatment, compared to non-tumor bearing mice. (E) Average BV/TV were assessed using microCT analysis of tibiae collected from 4 week old athymic nude mice mouse treated with either vehicle or doxorubicin showing loss of trabecular bone volume (*P* = 0.02). (F) Representative X-ray images showing bone loss upon doxorubicin treatment, compared to control. Quantification of (G) osteolytic lesion area and (H) osteolytic lesion numbers in tumor-bearing Balb/C mice receiving 4T1 cell via cardiac injection and treated with either PBS or doxorubicin (5 mg/Kg) once per week for three weeks. (I) Ratlaps ELISA showing significant increase (*P*<0.05) in bone resorption upon doxorubicin treatment in non-tumor bearing mice. Statistical analysis was performed using student’s T–test. *P*<0.05 was considered significant. At least 5 mice were used in each group for these experiments.

An orthotopic model of 4T1-592 breast cancer bone metastasis was used to investigate doxorubicin bone loss (Figure 1A and 1B). Both tumor-bearing and non-tumor bearing mice have shown doxorubicin-mediated bone loss. To test whether treatment with doxorubicin may provoke additional bone loss in breast cancer bone metastases models, we have compared trabecular bone volume in age-matched non-tumor bearing and tumor-bearing animals, treated once a week either with PBS or doxorubicin (5 mg/kg body weight) for 4 weeks. MicroCT analyses of mouse tibiae were performed to assess bone loss in both non-tumor bearing and tumor bearing mice, following doxorubicin treatment (Figure 1C). A significant amount of bone loss was observed in non-tumor bearing animals (Figure 1D) upon doxorubicin treatment (*P* = 0.001). Bone loss was significantly amplified when the mice were injected with 4T1-592 tumor cells in the # 4 mammary fat pad of 4 week old female Balb/C mice (*P* = 0.0005). Both the non-tumor and tumor-bearing animals used in these experiments were of a congenic Balb/C background. Athymic nu/nu mice yielded a similar trend of bone loss following doxorubicin treatment (Figure 1E). When we used an intra-cardiac injection model using 4T1 cells, osteolytic lesions were detected at 4 weeks post-injection using radiographic images (Figure 1F). Our data indicate that doxorubicin treatment significantly (*P*<0.001) increased osteolytic lesion area and lesion numbers compared to the control group (Figure 1G, H), suggesting that treatment with doxorubicin further increases bone destruction in tumor-bearing mice.

To assess the direct effect of doxorubicin on bone resorption, we used RatLaps ELISA to measure bone-related degradation products from C-terminal telopeptides of type I collagen from the serum samples of the non-tumor bearing nu/nu mice treated with either PBS or doxorubicin (5 mg/kg) for 4 weeks. The average amount of RatLaps (ng/ml) in the doxorubicin treatment group was significantly higher (*P*<0.05) than in the control group (Figure 1I), suggesting that doxorubicin promoted bone resorption, even in the absence of a tumor in the bone microenvironment.

### Anti-TGFβ Antibody Treatment Improves Doxorubicin-mediated Inhibition of Osteoblast Differentiation and Increases the Frequency of Osteoblast Colony Forming Units

**Figure 2:**
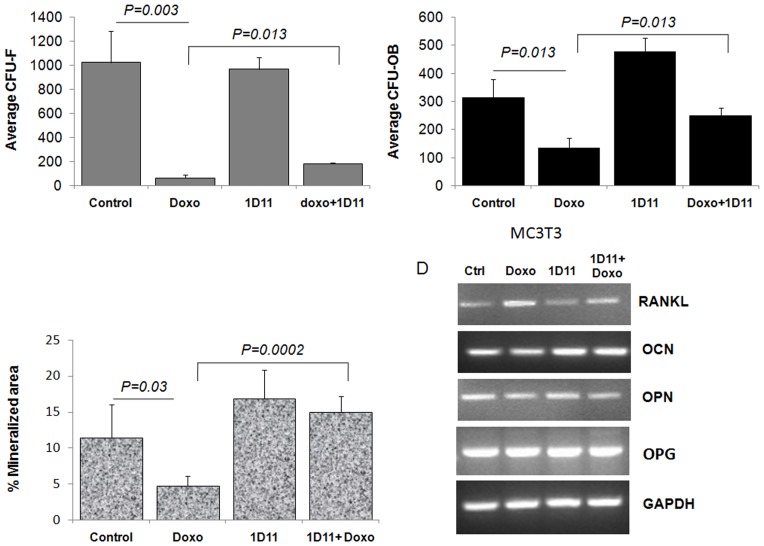
Anti-TGFβ antibody treatment improves doxorubicin-mediated inhibition of osteoblast differentiation and increases the frequency of osteoblast colony forming units. Mouse bone marrow cells were flushed and allowed to attach for two days. Bone marrow stromal cells were trypsinized and replated as 1×10^6^ cells per well in six well plates for fibroblast colony forming units (CFU-F) and 2×10^6^ cells for osteoblast colony forming units (CFU-OB). Cells were cultured either using DMEM-F12 media (10% FBS) alone or supplemented with either doxorubicin (0.01 ug/ml), 1D11(25 µg/ml) or a combination of both until fibroblast colonies were formed. CFU-OB were cultured using osteoblast differentiation media (alpha-MEM+10% FBS) containing ascorbic acid and β glycerophosphate with similar concentration of doxorubicin (0.01 µg/ml) and/or 1D11(25 µg/ml). Upon microscopic colony formation, media were aspirated, plates were washed in PBS, fixed with 10% neutral buffered formalin and stained to score (A) Average number of fibroblast colony forming units (CFU-F) per 1×10^6^ bone marrow cells. (B) Average number of osteoblast colony forming units (CFU-OB) per 2×10^6^ bone marrow cells. (C) Ex *vivo* osteoblast mineralization assay was performed using mouse calverial osteoblasts isolated from 3 days old pups and plated in triplicate. Upon confluence, cells were grown in osteoblast differentiation media containing ascorbic acid and β glycerophosphate, in presence of doxorubicin (0.01 µg/ml), 1D11(25 µg/ml) or a combination until mineralized matrix were formed. Von Kossa staining was performed as described in Materials and Methods and mineralization was scored using Metamorph software in each set and compared with media only group. Student T-test was performed to calculate p-values. P>0.05 was considered significant. N = 6 for each group was used in this experiment. (D) RT-PCR for expression of RANKL, OPG, OCN and OPN from MC3T3 cells treated with media alone, doxorubicin (0.01 µg/ml, 20 hours), anti-TGFβ antibody (25 ug/ml) and a combination of doxorubicin and 1D11.

Previous reports have indicated that doxorubicin treatment inhibits cell proliferation and parameters of cell differentiation in MC3T3 mouse osteoblasts [9] as well as reduced clonogenic ability and decreased osteoblast differentiation in cultured human mesenchymal stem cells [10], however the mechanism is not well understood. We have previously reported that doxorubicin treatment results in a systemic increase in the levels of TGFβ [11] and that blocking TGFβ may reverse pathological bone loss [19]. Here we tested whether treatment with the anti-TGFβ antibody 1D11 may protect bone marrow stromal cells and osteoblasts against doxorubicin-mediated damage. To determine this, bone marrow stromal cells were flushed from the long bones of non-tumor bearing C57BL/6J mice, plated at clonogenic density, and cultured for 15 days either in medium only, doxorubicin (0.01 µg/ml), 1D11 (25 µg/ml), or a combination of doxorubicin and 1D11. The medium used was appropriate to respectively support the growth of either fibroblast colony forming units (CFU-F) or osteoblast colony forming units (CFU-OB) culture as described under Materials and Methods. Our results indicate that treatment with doxorubicin causes a highly significant decrease in both fibroblast (*P* = 0.003) and osteoblast (*P* = 0.013) colony forming units and concurrent treatment with 1D11 was able to significantly improved numbers of both CFU-Fs (*P = 0.013*) and CFU-OBs (*P = 0.013*) (Figure 2A, B). Doxorubicin treatment affected the fibroblast population drastically by reducing the number of CFU-Fs by almost 90%, whereas number of CFU-OBs was reduced nearly 50%. Interestingly, treatment with 1D11 increased the number of CFU-OBs even higher than untreated control, suggesting a direct positive effect of anti-TGFβ antibody treatment on osteoblast colony forming units.

To investigate whether doxorubicin-mediated bone loss in non-tumor bearing mice is due to a detrimental effect of this chemotherapeutic agent on osteoblast mineralization, a crucial step for new bone formation, we used an *ex vivo* osteoblast mineralization assay. Osteoblast differentiation assay was performed using bone marrow stromal cells in presence doxorubicin (0.01 µg/ml), 1D11(25 µg/ml) or a combination of both as described in the Materials and Methods section. Doxorubicin treatment significantly (*P* = 0.03) decreased the osteoblast mineralization compared to control (Figure 2C). Treatment with 1D11 increased osteoblast mineralization compared to untreated controls, which was consistent with our earlier report [19]. Taken together, our data indicate that treatment with the anti-TGFβ antibody 1D11 may protect against doxorubicin-mediated damage by promoting osteoblast survival and differentiation.

Consistent with decreased osteoblast differentiation, an increase in RANKL expression was noted in MC3T3 cells upon treatment with doxorubicin which was restored to normal level following 1D11 treatment (Figure 2D), while, the level of OPG remained unaltered. In addition, treatment with doxorubicin has decreased the expression of both OPN and OCN in MC3T3 cells (Figure 2D).

### Doxorubicin Increases Osteoclast Formation

**Figure 3:**
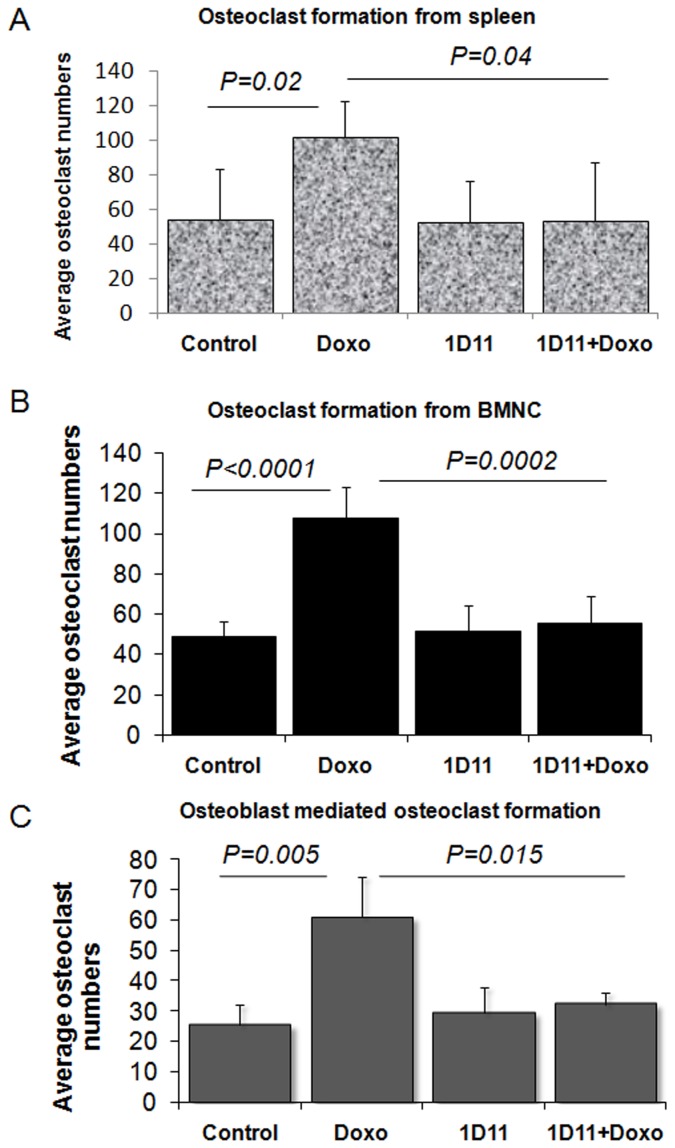
Anti-TGFβ antibody inhibits doxorubicin-mediated increase in osteoclast formation. Mononuclear cells from spleen (A) and bone marrow (B) from C57BL/6 were isolated and cultured for 15 days in the presence of MCSF and RANKL until mature osteoclasts are formed and scored using TRAP staining as described in Materials and Methods. (C) Osteoblast-mediated osteoclast formation was also done using a co-culture system as described in Materials and Methods. Student T-test was performed and P<0.05 was considered significant.

*Ex vivo* osteoclast formation was performed as described under Materials and Methods. In brief, bone marrow mononuclear cells were isolated from either spleen or bone marrow of adult mice and cultured using osteoclast differentiation media alone or containing (0.01 µg/ml) doxorubicin, (25 µg/ml) 1D11, or a combination of both. Upon doxorubicin treatment, a significant increase in average osteoclast numbers was noted in cultures from both spleen (Figure 3A, *P* = 0.02) and bone marrow mononuclear cells (Figure 3B, *P*<0.0001). Interestingly, 1D11 was able to decrease osteoclast numbers to the basal level. Moreover, 1D11 treatment alone did not show any change in the osteoclast numbers.

Osteoblasts and preosteoblasts maintain the homeostasis of osteoclasts in bone microenvironment by secreting both OPG and RANKL [36], [37], [38], [39], [40]. To test whether treatment with doxorubicin may affect osteoblast-mediated osteoclast formation, we exploited a co-culture assay system. Bone marrow mononuclear cells and calvarial osteoblasts from adult non-tumor bearing C57BL/6J mice were used in this experiment, as described in the Materials and Methods section. Our result showed a statistically significant increase in osteoblast-mediated osteoclast numbers after treatment with doxorubicin treatment (*P* = 0.005), and 1D11 treatment restored the osteoclast numbers to a normal level (Figure 3C). This supports our previous finding that treatment with doxorubicin probably induces TGFβ, which in turn modulates the RANKL/OPG ratio and favors osteoclast formation, both directly and indirectly.

### Anti-TGFβ Antibody Treatment Rescues Doxorubicin-mediated Bone Loss in Preclinical Breast Cancer Bone Metastasis Models

**Figure 4:**
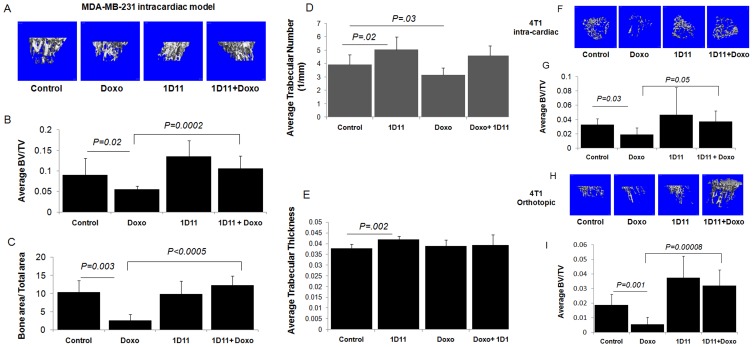
Anti-TGFβ antibody rescues doxorubicin mediated bone loss in breast cancer bone metastasis. MDA-MB-231 cells (1×10^6^) were inoculated via left cardiac ventricle of four-week old female athymic nude mice and treated with PBS, doxorubicin (5 mg/Kg, once weekly for 4 weeks, i.p.), 1D11 (10 mg/Kg, three times per weekly for 4 weeks) and a combination of doxorubicin and 1D11 for three weeks. (A) Representative microCT images of mice tibiae from each treatment group (B) Quantification of trabecular bone volume (BV/TV), (C) Histology of mice tibia from each group revealed trabecular bone loss upon doxorubicin treatment, both of which were rescued upon anti-TGFβ antibody treatment. Quantification of (D) average trabecular numbers and (E) average trabecular thickness. Representative microCT images from tumor-bearing mice in which (F) 4T1 cells (10^5^) were inoculated either via intracardiac route or (H) in the #4 mammary fat pad of four-week old female Balb/c mice which received either PBS, doxorubicin (5 mg/Kg, once weekly for 4 weeks, i.p.), 1D11 (10 mg/Kg, three times per weekly for 4 weeks) or a combination of doxorubicin and 1D11 for three weeks. Quantification of microCT images show significant loss of trabecular bone volume (BV/TV) in both intracardiac (G) and orthotopic (I) models. At least 5 mice were assessed in each group and P<0.05 was considered significant.

We have previously reported that doxorubicin increases circulating levels of TGFβ, a prometastatic cytokine implicated as a major driver of the vicious cycle of bone metastases. We hypothesized that, treatment with doxorubicin will increase bone loss in our preclinical models in a TGFβ dependent manner. MicroCT analyses of mouse long bones revealed that doxorubicin treatment resulted in significant decrease in trabecular bone volume (measured as BV/TV) in all tumor-bearing mice compared to untreated control. Bone loss in these mice was rescued by the anti-TGFβ antibody treatment (Figure 4B, G, I). Histological analysis also indicated that, in mice injected with MDA-MB-231 cells, trabecular bone area was significantly decreased (Figure 4C, *P* = 0.003) following treatment with doxorubicin and 1D11 was able to rescue bone area. In the MDA-MB-231 cardiac injection model, further analysis using quantitative microCT showed a statistically significant decrease in number of trabeculae upon doxorubicin treatment compared to the control group, which was restored to the normal level upon treatment with 1D11 (Figure 4D). Our treatment with doxorubicin did not affect the trabecular thickness as such, however, 1D11 treatment has generally improved trabecular thickness, underscoring its potential as a bone anabolic agent (Figure 4E). Interestingly, doxorubicin treatment resulted in an increase in the tumor area in the trabecular bone, which was decreased by 1D11 (Figure S1).

### Doxorubicin Mediates Bone Damage via Increased Oxidative Stress

**Figure 5:**
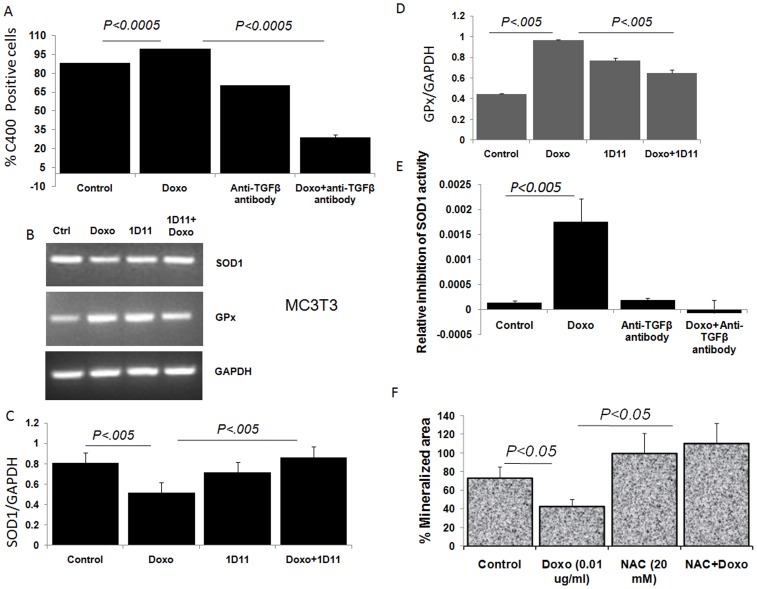
Doxorubicin mediates bone loss by elevating oxidative stress. (A) C400 oxidation show increase in reactive oxygen species (ROS) upon *in vitro* doxorubicin (0.01 ug/ml, 20 hours) treatment increases oxidative stress in the mouse bone marrow stromal cells, which was decreased by concomitant treatment with 1D11(25 µg/ml). Data represents average percentage of C400 positive cells from triplicate samples. (B) RT-PCR showing a decrease in SOD1 (copper zinc superoxide dismutase 1) and GPx expression was noted in MC3T3 mouse osteoblast cells upon treatment with doxorubicin (0.01 µg/ml, 20 hours), which was returned to normal level by co-treatment with anti-TGFβ antibody 1D11(25 ug/ml). (C) SOD1 expression normalized against GAPDH expression, quantified by Image J. (D) GPx expression normalized against GAPDH expression, quantified by Image J. (E) SOD1 activity was performed using MC3T3 cells as described in Materials and Methods section. The inhibition of SOD1 activity was measured by formation of NBT-diformazan from NBT following 20 hours treatment in either serum free alpha-MEM media alone, or supplemented with 0.01 ug/ml doxorubicin, 25 µg/ml anti-TGFβ antibody and a combination of both doxorubicin and anti-TGFβ antibody. A drastic inhibition of SOD1 activity was noted following doxorubicin treatment which was restored by anti-TGFβ antibody. (F) Calvarial osteoblasts from wild type mice (3–4 days old pups) were cultured until confluent and treated with osteoblast differentiation media supplemented with doxorubicin (0.01 µg/ml), N-acetyl cysteine (NAC, 20 mM) treatment, or a combination of both, or media alone until mineralized matrix was formed. Quantification of Von Kossa staining images from at least 3 different fields were done using Metamorph software.

Increased and persistent oxidative stress has been associated with doxorubicin-mediated cardiotoxicity [41]. To determine whether treatment with doxorubicin also increases oxidative stress in the bone marrow stromal cells, uptake of C400 measured as described in the Materials and Method section. Treatment with doxorubicin significantly (*P*<0.0005) increased the number of C400 positive cells suggesting an increase in oxidative stress upon doxorubicin treatment (Figure 5A, and histogram presented in Figure S2) and treatment with anti-TGFβ antibody was able to suppress oxidative stress below the basal level.

Furthermore, following doxorubicin treatment, MC3T3 bone marrow stromal cells have shown decreased SOD1 gene expression in a TGFβ mediated manner (Figure 5B). SOD1 is a major antioxidant defense enzyme implicated in tolerance of oxidative stress [42]. In addition, an increase in GPx gene expression (Figure 5B, C, D) also accompanied doxorubicin treatment, whereas GST expression remained unaltered (data not shown).

In addition, inhibition of SOD1 activity was noted in MC3T3 cell followed by doxorubicin treatment, which was improved when cells received a combined treatment with doxorubicin and anti-TGFβ antibody (Figure 5E). Increased oxidative stress has been negatively associated with osteoblasts differentiation and survival [23], [24]. We hypothesize that treatment with an anti-oxidant agent may be able to rescue doxorubicin-mediated suppression of osteoblast differentiation. Consistent with that, we were able to rescue doxorubicin-mediated suppression of osteoblast differentiation (Figure 5F) by concurrent treatment with N-acetyl cysteine (NAC), a known antioxidant agent, suggesting that doxorubicin-mediated bone loss may be in part is mediated via increased oxidative stress.

## Discussion

Doxorubicin is a widely used anthracycline drug for patients suffering from primary as well as metastatic breast cancer [3] and has an overall response rate of up to 50%. With current therapeutic options, patients with metastatic disease show a median survival of about 2 years. However, the enthusiasm over usage of doxorubicin has dampened due to a number of side effects such as myelosuppression, immunosuppression and cardiotoxicity [43]. In addition, preclinical and clinical reports from patients with childhood cancers have indicated significantly disruptive side effects on the skeletal system including increases in pathologic fracture [44], [45], reduced bone mineral density, diminished bone formation [7], and compromised adult height. Despite several reports of the damaging effect of doxorubicin on bone cells, not much was known about whether treatment with this agent may accelerate bone loss in breast cancer patients, who are already suffering from osteolytic disease.

In the context of metastatic breast cancer, when the cancer cell reaches the bone microenvironment, an increase in osteoclastic bone resorption takes place. As a result, an excess of active TGFβ is secreted in the bone microenvironment, which in turn mediates a cascade of events that favor the vicious cycle of bone metastasis [46]. In addition, TGFβ increases osteoclast differentiation [47], [48], and suppresses osteoblast differentiation [49], all of which may contribute to the accelerated bone destruction. We have previously reported that treatment with doxorubicin can increase circulating levels of TGFβ in both normal and tumor-bearing mice [11]. In addition, we and others have shown that blocking TGFβ either by antibodies or small molecules can prevent metastatic progression in the bone [19], [50] and that suppression of TGFβ improves the overall bone volume in normal and tumor-bearing animals [20], [51]. Interestingly, Bandyopadhyay et al. [52] have recently reported that combined treatment doxorubicin and a small molecule inhibitor of TGFβ receptor kinase [53], [54] improved doxorubicin’s efficacy in the inhibition of breast cancer growth and metastasis. Previously, Bandyopadhyay et al have also reported an increase in metastatic incidence and tumor load in the bone using the 4T1 model [52]. We also observe a similar trend in our study (Figure S1). We anticipate that the increase in tumor incidence following doxorubicin treatment may have been an understudied area. However, as doxorubicin increases TGFβ in the circulation, it is not unlikely that doxorubicin regimen may negatively affect tumor load when it has spread to the bone. Further clinical studies are needed to assess the bone metastases in the patient population receiving doxorubicin.

Chemotherapy can induce bone loss both directly [55] and indirectly due to ovary failure. Apart from its indirect effects on bone, several studies have indicated that doxorubicin exposure directly affects matrix production by osteoblasts and decreases osteoid volume [56]. Trabecular bone volume was also reduced in doxorubicin-treated normal rat [7]. In agreement with these previous findings, we found that doxorubicin treatment can significantly increase bone loss in preclinical models of breast-to-bone metastasis. In addition, loss of trabecular bone volume and an increase in bone resorption markers was also noted in non-tumor bearing mice, suggesting a tumor-independent effect of doxorubicin on the bone microenvironment. At the cellular level, doxorubicin treatment decreased osteoblast differentiation when used *in vitro*. We anticipate that *in vivo* treatment with doxorubicin may also decrease stromal and osteoblast cell survival and disrupt bone homeostasis. This is in agreement with the previous report by Guest et al. [57] showing that, a single dose of doxorubicin (10 mg/kg) has completely inhibited the stromal cell population in rat. Consistent with bone loss, an increase in RANKL and decrease in both OPN and OCN were noted in MC3T3 cell line upon treatment with doxorubicin. Treatment with anti-TGFβ antibody can decrease the RANKL induction to a normal level and restore OPN and OCN expression. Of note, in a clinical study, Jacot et al reported a decline in vitamin D levels along with decrease in soluble RANKL [58] in a patient cohort following adjuvant chemotherapy. We believe our finding captures a change in the cellular (osteoblast) level and increased RANKL may be a reflection of decreased osteoblast differentiation, as undifferentiated osteoblasts secrets higher amount of RANKL compared to fully differentiated osteoblasts. The publication by Jacot captures a much complex scenario in patient population which may be a direct effect of vitamin D insufficiency on the overall physiology and bone markers in a cohort of locally advanced breast cancer patients. In addition, decrease in RANKL may indicate an overall decrease in the number of osteoblasts, which was not quantified in this study. Jacot et al [58] also reported that the patients received epirubicin as a part of their regimen whereas, we have used doxorubicin, so the direct effect on each agent may vary as well. Another study using rodent models have indicated that depletion of vitamin D leads to increased RANK:OPG ratio [59], suggesting further study will be needed to identify the relationship between vitamin D levels and RANKL. However, our finding agrees with the overall bone loss and decreased osteoblast differentiation in our model. Interestingly, increased TGFβ has been reported to suppress OCN transcription and osteoblast differentiation *in vitro* and *in vivo* [60]. OPN was also a positive regulator of bone formation and osteoblast differentiation [61], [62].

In addition, *ex vivo* culture of bone marrow mononuclear cells have revealed that doxorubicin treatment increases osteoclast differentiation. However, when histological analysis was performed, the number of osteoclasts also decreased in the doxorubicin treated tumor-bearing mice, when compared with untreated tumor-bearing mice. We attribute the latter finding to severe damage of the bone marrow progenitor population, as previous reports have speculated that long term doxorubicin treatment results in a cytotoxic action on both osteoblasts and osteoclasts [8]. Using a co-culture assay system, we have also shown that doxorubicin treatment increased osteoblast-mediated osteoclastogenesis. This emphasizes an indirect yet very important role for osteoblasts in affecting doxorubicin-induced osteolytic bone damage.

We have demonstrated that treatment with doxorubicin can severely suppress osteoblast differentiation, and anti-TGFβ antibody treatment can restore this to normal level. Of note, doxorubicin administration was shown to decreased bone formation in young rat [7]. In addition, both the stromal and osteoblast progenitor survival were also disrupted upon doxorubicin treatment, suggesting long-term damage in the bone marrow microenvironment.

One of the objectives of this study was to identify a molecular mechanism by which doxorubicin accelerates bone loss. Increased TGFβ has been implicated in increased production of ROS. Our data indicate that, treatment with doxorubicin increases reactive oxygen species in MC3T3 osteoblast cells, which was accompanied by a decrease in SOD1 expression. In addition, following doxorubicin treatment, an inhibition of SOD1 enzyme activity was also noted which was rescued by anti-TGFβ antibody treatment. Interestingly, it has been recently reported that loss of SOD1 decreases osteoblast viability and induces bone loss in mice [63]. Smietana et al have reported that loss of SOD1 resulted in weaker bone and lower bone mineral density in mice [64]. SOD1 has been reported to restore osteoblast differentiation by attenuating oxidative stress in human dental pulp cells [65], which is in agreement with our result that treatment with antioxidant agent NAC was able to rescue doxorubicin-mediated decrease in osteoblast differentiation. An inverse relationship was noted between TGFβ1 and SOD1 activity in radiation-mediated fibrosis models [66], [67].

Since doxorubicin is known to significantly increase oxidative stress, we suspect other antioxidant enzyme may also play a role in the tissue injury mediated by this agent. In agreement with this, an increase in the GPx level was noted in MC3T3 cells upon doxorubicin treatment, with no change in catalase and HO1 levels. Earlier studies have indicated an increase in GPx activity in elderly people who exhibit higher oxidative stress due to natural aging [68] and GPx level was increased following doxorubicin treatment in human lymphoblastoma cell line (with functional p53) [69], whereas catalase level was not increased.

In conclusion, our data indicate, treatment with doxorubicin deregulates multiple pathways in bone marrow stromal cells and blocking TGFβ may rescue those events and prevent doxorubicin mediated bone loss. While doxorubicin-mediated oxidative stress have been implicated in cardiotoxicity, to our knowledge, this is the first report of SOD1 being the molecular target of doxorubicin-mediated bone damage. Based on this finding and those that indicate that TGFβ blockade can prevent metastatic progression to bone, we conclude that treatment with anti-TGFβ antibody 1D11 may have a two-fold benefit for the breast cancer patients by both inhibiting cancer progression and preventing bone loss. We anticipate this finding will lead to improved management and patient care for breast cancer survivors who have received doxorubicin as a part of their treatment regimen.

## Materials and Methods

### Study Design

Tumor bearing and non-tumor bearing mice were treated using 5 mg/kg doxorubicin (Sigma: Doxorubicin hydrochloride, D1515), once weekly for three weeks. For *in vivo* rescue experiments related to anti-TGFβ treatment, both the control antibody and the anti-TGFβ antibody (1D11) were obtained from Genzyme corporation. 1D11 blocks all three isoforms of TGFβ [19]. To test the efficacy of anti-TGFβ antibody in preventing doxorubicin-mediated bone loss in both intracardiac and orthotopic model of breast cancer metastasis to bone. Mice were treated with anti-TGFβ antibody (1D11, 10 mg/kg), starting either 1 day (intracardiac) or 1 week (orthotopic) after tumor cell inoculation; treatment frequency was 3 days per week. The vehicle used for preparing the antibodies showed no significant difference compared to the control-antibody-treated group during initial experiments and was therefore excluded from these studies (communication with Genzyme Corporation). The outcome measures included quantification of trabecular bone volume (BV/TV), trabecular numbers and trabecular thickness from microCT of tibiae and histological analyses. Animals were sacrificed after three weeks and within this pediod we have not seen any drastic issue with survival. However, a expected, mice treated with doxorubicin had a rapid weight loss compared to other groups. We have measured body weight of the animals every other day throughout the study. Our animal care regulation dictates that we sacrifice the mice if any treatment lead to more that 10% weight loss. All mice which were alive after three weeks were used for the analysis. We have seen occasional death of mice before three weeks and those mice were excluded from the analysis.

### Cell Culture

Bone metastatic variant of MDA-MB-231 was generated as described earlier [19]. In brief, MDA-MB-231 human breast cancer cell line was obtained from ATCC (American Type Culture Collection) and a bone metastatic variant generated previously in the group was used for all *in vitro* and *in viv*o studies [70]. The murine mammary cell line 4T1 had previously been obtained from another investigator [71] and used in a cardiac injection model within our group. Both cell lines were maintained in DMEM (Invitrogen, Carlsbad, CA) containing 10% Fetal Bovine Serum (FBS: Hyclone Laboratories, Logan, UT) and 1% penicillin/streptomycin (Mediatech). Cells were cultured in a 37°C atmosphere of 5% CO_2_ and 95% O_2_ using standard tissue culture techniques. 4T1-592 cells were generated as described earlier and we have received it from a collaborator Dr. F Elefteriou, Director, Center for Bone Biology, Vanderbilt University [72], [73].

### Animals

All procedures were performed with the approval of the Vanderbilt University Institutional Animal Care and Use Committee and in accordance with Federal guidelines. For all *in vivo* experiments, 4- to 5-week-old female athymic nu/nu mice (for MDA-MB-231 human breast cancer cells) or Balb/C mice (for 4T1 mouse mammary tumor cells) were used. Both athymic nu/nu mice and Balb/C mice were purchased from Harlan Laboratories.

### Intra-cardiac Bone Metastasis Model

Intra-cardiac injection was performed as described earlier [19]. MDA-MB-231 or 4T1 cells were trypsinized, washed and then resuspended in ice-cold sterile PBS at a final concentration of 1×10^6^/ml. Four- to five-week-old female nude or Balb/C mice were anesthetized using a ketamine/xylazine mixture. Mice were positioned ventral side up, and tumor cells were injected into the left cardiac ventricle using a percutaneous approach with a 27-gauge needle, as described previously [74], [75]. Each mouse received 1×10^5^ cells in a 100-µl volume (resuspended in PBS). Treatment was started either from the next day (intra-cardiac) or 1 week(mammary fat pad injection) following tumor cell injection. Mice were imaged weekly and sacrificed as described under each experiments. Any mice showing signs of distress were sacrificed immediately.

### Orthotopic Bone Metastases Model

For orthotopic breast cancer to bone metastases model, 4T1-592, 50 µL of a PBS solution containing 5×10^3^ cells were injected into the 4^th^ mammary fat pad of 4 week old female Balb/C mice. After 1 week, palpable tumor was noticed and treatment with doxorubicin and/or antibodies were started.

### Colony Formation Assays

Bone marrow cells were collected from the tibiae and femora of adult C57BL/6 mice using a PBS and antibiotic mixture (2000 U/ml penicillin and 2000 U/ml streptomycin). Cells were washed in 250 g for 5 minutes and supernatant was aspirated. Cells were resuspended in media (α-MEM, 10% FBS, 2000 U/ml penicillin, 2000 U/ml streptomycin, and 250 µg/ml fungizone) and plated for at least 48 hours. After 48 hours, unattached cells were aspirated and attached cells were trypsinized, resuspended in single cell suspensions, counted and plated in appropriate cell density for colony formation assays. The fibroblast colony forming unit (CFU-F) assay was done using 10^5^ cells per well and the osteoblast colony forming unit (CFU-OB) assay with 2×10^5^ cells per well in a 6-well plate as previously described [76]. Cells were allowed to attach overnight and treatment with doxorubicin and/or 1D11 were started from one day after the cells were plated. Cells were treated with media supplemented with doxorubicin (0.01 µg/ml), 1D11 (25 µg/ml), or a combination of both. A control set was cultured using media alone. After visible colonies were noted, cells were fixed by 10% formalin and stained with 0.5% crystal violet for CFU-F and with Von Kossa for CFU-OB assays. Numbers of colonies were counted under a microscope.

### Osteoblast Mineralized Matrix Formation Assay

Calvarial osteoblasts were isolated from 3- to 4-day-old mice pups, using a modification of a sequential collagenase/trypsin digestion method [77]. In brief, calvaria were removed from 3- to 4-day-old C57BL/6J mice. Soft tissues were cleaned, washed for 10 minutes with PBS containing 0.025% trypsin, digested with 10 mg/ml type-IV collagenase *p* (*Clostridium histolyticum*, Roche) in α-MEM, and incubated for 30 min at 37°C with gentle shaking. The procedure was repeated twice, with a 1-hour digestion followed by a 30-minute digestion. The cells from the second and third digestions were collected and centrifuged at 2500×g for 10 min. The media were aspirated and discarded, and the pellet was resuspended and plated in α-MEM containing 10% FBS. The culture was kept undisturbed for at least 2 days. At confluence, cells were trypsinized and plated in 6-well plates for the osteoblast differentiation assay. Cells were cultured until confluence, at which time doxorubicin treatment (0.01 µg/ml) and/or 1D11 treatment (25 µg/ml) was performed. For the rescue experiment, cells were treated with 1D11, 30 minutes prior to doxorubicin treatment. Following the treatment, cells were cultured in osteogenic medium, which was prepared using α-MEM containing 10% FBS, 5 mM β-glycerophosphate (MP biochemical) and 50 g/ml L-ascorbic acid (Sigma) with changes every 2 days until mineralized nodules are formed (approx. 15–21 days). Mineralized matrix formation was detected by Von Kossa staining, and photomicrographs were taken.

### Co-culture Assay

*Ex vivo* co-culture assays were done using mouse calvarial osteoblasts and adult mouse bone marrow mononuclear cells. Calvarial osteoblasts were isolated from 3- to 4-day-old pups (C57BL/6J) following the method described earlier [77] and cultured in 24-well tissue culture plates until confluent. After the osteoblast culture reached confluence, bone marrow mononuclear cells were isolated from adult mice and plated on top of the osteoblast layer. The co-culture system was treated with doxorubicin (0.01 µg/ml) and 1D11 (25 µg/ml) every other day for 7–10 days. Cells were fixed and stained for assessment of mature osteoclast formation using a Leucocyte Acid Phosphatase kit (Sigma) according to the manufacturer's instructions, and mature osteoclasts (red) were counted using a microscope.

### Osteoclastogenesis Assays

Osteoclast differentiation assays were performed as reported previously [19]. Briefly, mouse long bones were flushed with PBS, resuspended by pipetting, and strained through a 40 µM cell strainer (BD Biosciences, San Jose, California). Bone marrow mononuclear cells were isolated using Histopaque 1077 (Sigma) following the manufacturer’s instructions and cultured for 7–10 days in α-MEM supplemented with 10% FBS, 100 ng/ml RANKL (R&D systems) and 30 ng/ml macrophage colony stimulating factor (MCSF; R&D systems) to support osteoclast differentiation. TRAP staining was performed using a Leukocyte Acid Phosphatase kit (Sigma, St Louis, MI) and the number of osteoclasts per field was counted under a microscope.

### Measurement of Cellular ROS by Flow Cytometry

Femora and tibiae were collected from adult C57/BJ mice and bone marrow cells were washed using sterile PBS and antibiotic (2000 U/ml penicillin and 2000 U/ml streptomycin), resuspended (α-MEM, 10% fetal bovine serum (FBS), 2000 U/ml penicillin, 2000 U/ml streptomycin, and 250 µg/ml fungizone), plated in tissue culture flasks and returned to a cell culture incubator (37°C, 5% CO_2_). After 2–3 days, non-adherent cells were removed and adherent cells were collected separately and used for ROS assays using 2,7-dicholorofluorescein diacetate (H2DCF-DA, Molecular Probes) according to the manufacturer’s instructions. In brief, cells were treated with either media alone or supplemented with doxorubicin (0.01 µg/ml) for 20 hours. Next day, cells were trypsinized, washed and resuspended in PBS, combined with the molecular probe C400 (10 mg/ml) and incubated for 15 min at 37°C. Cells were analyzed using flow cytometry for C400 oxidation in the Vanderbilt University flow cytometry core [78].

### RatLaps ELISA

To assess bone resorption, quantitative determination of bone-related degradation products from C-terminal telopeptides of type I collagen were measured using a commercially available ELISA assay kit (ImmunoDiagonosticSystems, Catalog # AC-06F1) following the manufacturer's guidelines. In brief, blood samples were collected from all animals on the same day (between 9∶00 and 10∶00 AM) at the end of the experiment and sera were prepared, as measuring the type I collagen fragments for *in vivo* models of bone disease, in order to avoid variability among samples. Serum samples were stored at −20°C, until the ELISA assay was performed. At least 9 samples per group were assessed.

### RNA Isolation, cDNA Synthesis, and RT-PCR

MC3T3 murine osteoblast cell lines were cultured until 75% confluence and then treated either with media alone, doxorubicin (0.01 µg/ml), 1D11 (25 µg/ml) and a combination of doxorubicin and 1D11. Cells were harvested after 20 hours, washed by PBS and used for RNA isolation and cDNA preparation as described earlier [79]. Specific PCR conditions and primer design for OPG, RANKL and GAPDH were described earlier [79]. In brief, RANKL and GAPDH were amplified using 94°C for 2 minutes followed by 35 cycles of 94°C for 15 seconds, 60°C for 30 seconds, 72°C for 45 seconds annealing, and a final extension of 72°C for 10 minutes. For OPG, PCR conditions were 94°C for 2 minutes followed by 35 cycles of 94°C for 15 seconds, 57°C for 30 seconds, 72°C for 45 seconds annealing, and a final extension of 72°C for 10 minutes. For SOD1 and GPx amplification [80], PCR conditions were 94°C for 2 minutes followed by 25 cycles of 94°C for 15 seconds, 57°C for 30 seconds, 72°C for 45 seconds annealing, and a final extension of 72°C for 10 minutes. Using methods described by Gevorgyan et al [81] OCN (bone Gla protein) was amplified using 94°C for 2 minutes followed by 25 cycles of 94°C for 1 minute, 58°C for 45 seconds, 72°C for I minute annealing, and a final extension of 72°C for 10 minutes. Similarly, secreted phosphoprotein 1(OPN) was amplified using 94°C for 2 minutes followed by 18 cycles of 95°C for 1 minute, 55°C for 2 minutes, 72°C for I minute annealing, and a final extension of 72°C for 10 minutes [81]. The PCR products were electrophoresed in 2% agarose gels. Images were captured using a BioRad gel documentation system and band intensities were quantified using Image J software. The following sets of primers were used for RT-PCR:

GPx-1∶5′CACAGTCCACCGTGTATGCCTTCT- CCA GGAACTTCTCAAAGT3’ (500 bp)

Cu-ZnSOD (SOD1): 5′GGGAAGCATGGCGATGAA- GCAGATGAGTCTGAGACTCAGA3’ (500 bp)

OPG,5′-GTGGTGCAAGCTGGAACCCCAG-AGGCCCTTCAAGGTGTCTTGGTC-3′ (647 bp);

RANKL,5′-CGCTCTGTTCCTGTACTTTCGAGCG-CGTGCTCCCTTTCATCAGGTT-3′ (587 bp);

OPN, 5′ TCACCATTCGGATGAGTCTG-ACTTGTGGCTCTGATGTTCC3’, (36 bp)

OCN, 5′ CCTCAGTCCCCAGCCCAGATCC- CAGGGCAGAGAGAGAGGACAGG3’ (219 bp)

GAPDH: 5′-CTGCACCACCAACTGCTTAG-AGATCCACGACGGACACATT -3′ (282 bp);

### SOD1 Activity

Effect of doxorubicin on SOD1 enzyme activity was measured in MC3T3 cells using Superoxide Dismutase assay kit (Trevigen, Catalog # 7500-100-K). In brief, cells were plated in T75 flasks and treated for 20 hours in either serum free alpha-MEM media alone, or supplemented with 0.01 µg/ml doxorubicin, 25 µg/ml anti-TGFβ antibody and a combination of both doxorubicin and anti-TGFβ antibody. Next day, both floating and attached cells were collected and centrifuged at 300 g for 10 minutes. Media was aspirated and cells were briefly washed using ice cold phosphate buffered saline using previous centrifugation speed. The pellet was resuspended using 10 volume lysis buffer, transferred to an Eppendorf tube and placed in an incubator for 30 minutes with periodic vortexing followed by centrifugation at 10,000 g for 10 minutes in a refrigerated Eppendorf centrifuge. The supernatant was transferred to a fresh pre-chilled Eppendorf tube and subjected to chloroform ethanol extraction. The upper aqueous phase was used for SOD1 activity assay. The inhibition of SOD1 activity was measured by formation of NBT-diformazan from NBT by colorimetric assay.

### Statistical Analysis

Student’s T-test was used to determine the statistical significance of the results. A *P*-value <0.05 was considered statistically significant. At least 5–6 mice per group were used for each *in vivo* and *in vitro* experiment. All *in vitro* experiments using cell lines were performed at least three times.

## Supporting Information

Figure S1 **Histological sectioned stained with H&E were used or tumor area assessment.** Images were taken and were quantified using Metamorph software. At least five animals were in each group. A ratio of trabecular area versus tumor area was used to generate the ratio.
(TIF)

Figure S2 **Unstained cells were used to measure for autofluorescence in the green emission range.** All other cells were stained using the protocol outlined in Materials and Methods section. Panel A compares the unstained cells and control cells which were positive for C400 uptake (87.9%). Panel B represents unstained cells with C400 positive cells treated with doxorubicin (99%). Panel C represents unstained cells with C400 positive cells treated with anti-TGFβ antibody (71.2%). Panel D represents unstained cells with C400 positive cells treated simultaneously with doxorubicin and anti-TGFβ antibody (26.8%).
(TIF)
