# Correction: Doxorubicin-Mediated Bone Loss in Breast Cancer Bone Metastases Is Driven by an Interplay between Oxidative Stress and Induction of TGFβ

**DOI:** 10.1371/annotation/95cefb34-2f3d-42a5-b73e-53c531591f0b

**Published:** 2013-11-12

**Authors:** Tapasi Rana, Anwesa Chakrabarti, Michael Freeman, Swati Biswas

In the Funding Statement, an organization was incorrectly as having provided funding for the article. The Funding Statement should read: "This work was supported by a Building Interdisciplinary Research Careers in Women's Health (BIRCWH) grant and Academic Project Support (APS) funding. The funders had no role in study design, data collection and analysis, decision to publish, or preparation of the manuscript." 

