# Supplementary figures and images for: Doxorubicin-Mediated Bone Loss in Breast Cancer Bone Metastases Is Driven by an Interplay between Oxidative Stress and Induction of TGFβ
Source: PLoS One. 2013 Oct 30;8(10):e78043. doi: 10.1371/journal.pone.0078043 (PMC3813496; doi:10.1371/journal.pone.0078043)

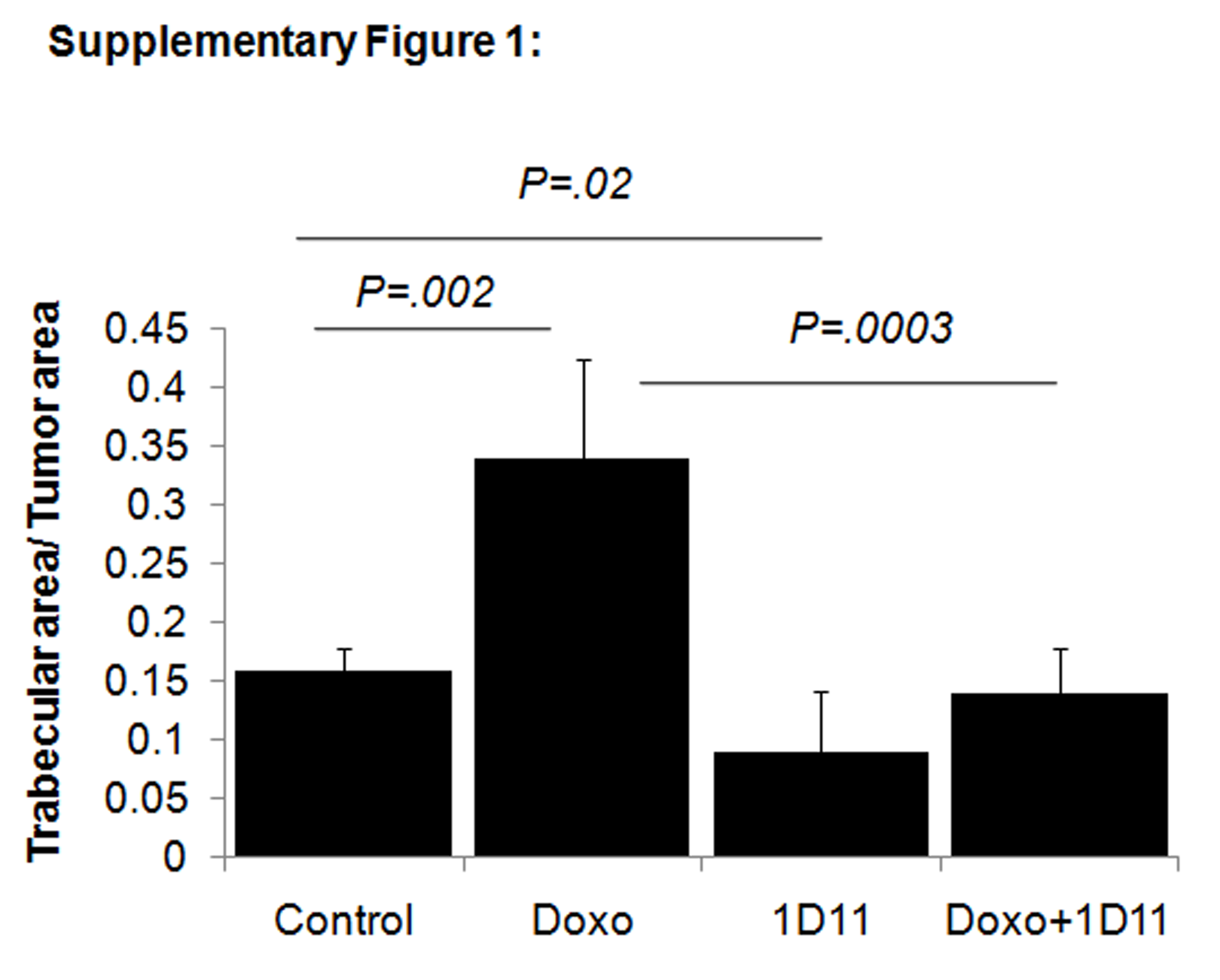

Supplement: Figure S1 — Histological sectioned stained with H&E were used or tumor area assessment. Images were taken and were quantified using Metamorph software. At least five animals were in each group. A ratio of trabecular area versus tumor area was used to generate the ratio. (TIF) [file pone.0078043.s001.tif]

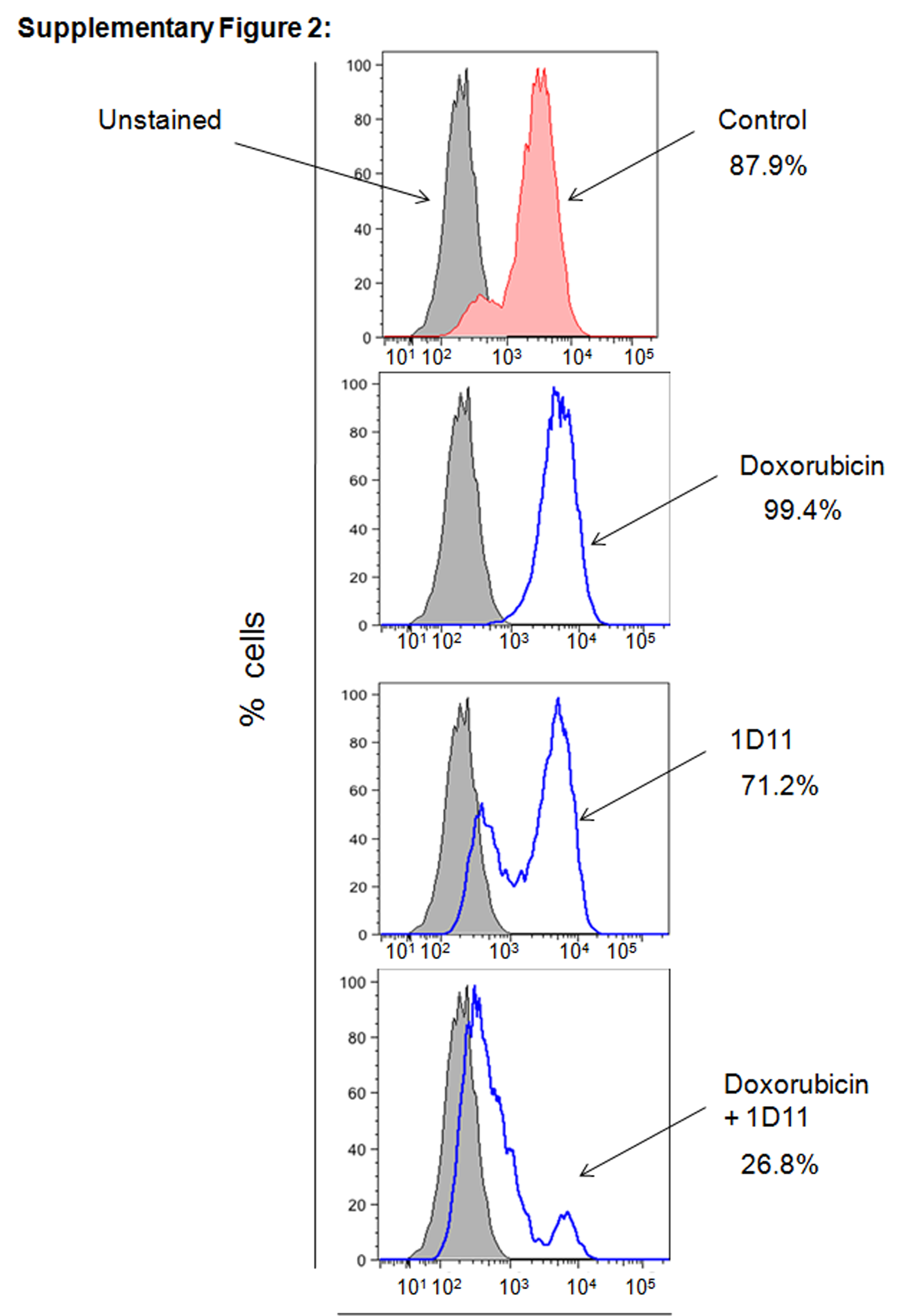

Supplement: Figure S2 — Unstained cells were used to measure for autofluorescence in the green emission range. All other cells were stained using the protocol outlined in Materials and Methods section. Panel A compares the unstained cells and control cells which were positive for C400 uptake (87.9%). Panel B represents unstained cells with C400 positive cells treated with doxorubicin (99%). Panel C represents unstained cells with C400 positive cells treated with anti-TGFβ antibody (71.2%). Panel D represents unstained cells with C400 positive cells treated simultaneously with doxorubicin and anti-TGFβ antibody (26.8%). (TIF) [file pone.0078043.s002.tif]
